# Role of Plants and Urban Soils in Carbon Stock: Status, Modulators, and Sustainable Management Practices

**DOI:** 10.3390/plants14040546

**Published:** 2025-02-10

**Authors:** Antonino Fiorentino, Farah Zahoor Rajput, Annamaria Di Serio, Vincenzo Baldi, Francesco Guarino, Daniela Baldantoni, Domenico Ronga, Pierluigi Mazzei, Oriana Motta, Mariarosaria Falanga, Angela Cicatelli, Stefano Castiglione

**Affiliations:** 1 Department of Chemistry, University of Milan, 20133 Milan, Italy; antonino.fiorentino@unimi.it; 2 National Biodiversity Future Center (NBFC), 90133 Palermo, Italy; frajput@unisa.it (F.Z.R.); adiserio@unisa.it (A.D.S.); vbaldi@unisa.it (V.B.); fguarino@unisa.it (F.G.); dbaldantoni@unisa.it (D.B.); dronga@unisa.it (D.R.); pmazzei@unisa.it (P.M.); omotta@unisa.it (O.M.); mfalanga@unisa.it (M.F.); scastiglione@unisa.it (S.C.); 3 Department of Chemistry and Biology “Adolfo Zambelli”, University of Salerno, 84084 Fisciano, Italy; 4 Department of Pharmacy, University of Salerno, 84084 Fisciano, Italy; 5 Department of Medicine Surgery and Dentistry, “Scuola Medica Salernitana”, University of Salerno, 84081 Baronissi, Italy; 6 Department of Information and Electric Engineering and Applied Mathematics, University of Salerno, 84084 Fisciano, Italy

**Keywords:** soil C cycle, urban soils, urban vegetation, biochar, compost, climate change

## Abstract

Urban soils are vital components of urban ecosystems, significantly influenced by anthropogenic activities and environmental factors. Despite misconceptions about their quality, urban soils play a pivotal role in carbon (C) cycling and storage, impacting global emissions and sequestration. However, challenges such as soil contamination, land use changes, and urban expansion pose significant threats to soil quality and C storage capacity. Over the last two decades, there has been an increasing interest in the C storage potential of soils as part of climate change mitigation strategies. In this review, a bibliometric analysis covering the last twenty years (2004–2024) was performed to offer insights into global research trends, mainly in urban soils of the Mediterranean region. This paper also identifies research gaps and proposes essential solutions for mitigating the negative impacts of urbanization on soil biodiversity and functions. Key modulators, including plants, microbes, and soil features, are highlighted for their role in C dynamics, emphasizing the importance of effective soil and vegetation management to enhance C sequestration and ecosystem services. Strategies such as reintroducing nature into urban areas and applying organic amendments are promising in improving soil quality and microbial diversity. Further research and awareness are essential to maximize the effectiveness of these strategies, ensuring sustainable urban soil management and climate resilience.

## 1. Introduction

Soils globally represent a primary carbon (C) sink for terrestrial ecosystems, storing approximatively four and half times more C than all living biomass, and more than three times as much as is present in the atmosphere [1]. The concentration of CO_2_ in the atmosphere, directly related to global warming [2], can be mitigated by sequestering carbon in terrestrial ecosystems, including soil, a concept first proposed in 1977 [3]. Since then, several studies have been performed on C capture and storage in the soil of different environments with a climate mitigation action (manipulation of soil C balance, C stock conservation strategies, long-term storage, etc.) [4]. Soil carbon (SC) content is determined by a complex equilibrium involving biological activity, between fixation (photo- and chemosynthesis) and release (respiration and decomposition) processes. This equilibrium is affected by several modulators including resources availability, climate and topography, edaphic communities, vegetation, and many others. Climate change itself can influence soil C dynamics. Global warning is leading to a general increase in average temperatures in many latitudes, and higher temperatures accelerate soil organic matter (SOM) decomposition, releasing more CO_2_. However, in some particular ecosystems, elevated temperatures and increased CO_2_ levels can stimulate plant growth, potentially leading to greater C inputs into the soil [5]. The development of policies and economic incentives to promote C sequestration in soils is crucial. These must include C trading schemes, subsidies for sustainable farming practices, and research funds for innovative soil management strategies. For example, conservative agricultural practices, such as crop rotation and the use of cover crops, can significantly enhance C sequestration in soils. Indeed, conservative agriculture, by reducing tillage and maintaining crop residues on the field, improves soil structure and increases its organic C (OC) content [6]. Similarly, sustainable management practices of urban soils need to be promoted.

The aim of this manuscript was to summarize scientific literature on the relevance, status, and threats on C soil, analyzing, based on the development timescale, both current and potential future technologies and methodologies for its sequestration and/or management. The literature review on this appealing topic was conducted according to a traditional narrative review. Due to the less stringent methodology, a traditional narrative review was considered by the authors to be quicker and easier to produce as compared to a systematic review, which requires a more structured and exhaustive approach and rigid methodological procedures. This choice was considered more advantageous because it allowed the authors—for a comprehensive exploration of the topic by including a wide range of quantitative and qualitative studies, without the restrictions of specific inclusion criteria—more flexibility to integrate, synthesize, and draw connections between different modulators of urban C soil, identifying research gaps and suggesting new strategies for the sustainable management of urban soils. At the same time, the authors tried to minimize subjectivity in the selection and interpretation of studies, addressing the intrinsic risk of over-simplification and avoiding potential bias in the findings of the literature search. Materials related to the topic (the C cycle in urban soils; the role of plants and other modulators on C stock; sustainable management practices in Mediterranean urban soils) were gathered using relevant databases and search engines, including Google Scholar, Scopus, Web of Science, and ResearchGate. The focus was on collecting a variety of studies from the last twenty years that provide a wide range of perspectives; combinations of the following terms were employed: urban soil, carbon soil sequestration, carbon soil sink, carbon soil storage, carbon soil handprint, microbial carbon sequestration, carbon fixing bacteria, biomineralization of carbon dioxide, biochar amendment, compost amendment, mineral fertilizers, microbially induced calcium carbonate precipitation, microbial carbonic anhydrase, urban vegetation, urban green, urban lawns, urban garden C sequestration, and urban forest carbon sequestration. Over 100 articles (research articles, review papers) were selected using some inclusion and exclusion criteria (English language, study design, journal relevance, Mediterranean area). The articles were examined, read, and their data (quantitative outcomes, key findings) summarized. Different modulators of C in urban soils, as well as technologies and methodologies for sequestration and/or management, were evaluated and classified into categories (such as microbial carbon sequestration, biochar and compost amendments, and mineral fertilizers); each category was further subdivided based on specific techniques and their applications in urban soil management.

## 2. Urban Soils: Features and Role in C Cycle

### 2.1. Definition and Features

The term ‘anthropogenic urban soil’ was coined by the geologist Ferdinand Senft (1847) and, from this definition, the concept of urban soils (USs) was derived [7]. In the following years, several definitions USs have been proposed by pedologists, even in relation to the influencing factors and taxonomical characteristics of the hosted community, also considering anthropogenic inclusions such as the following: transportation (road, railway networks), site disturbances (sealing of surfaces), buildings, intensity of use (trampling, hydraulic pressure), engineering interventions (green roofing, avenue plantations), etc. [8]. However, ambiguous concepts relative to USs still exist. In the 1960s, Zemlyanitskiy (1963) referred to the highly disturbed soils of urban areas as USs [9]. Later, Bockheim (1974) described USs as substrates having a non-agricultural destination, a man-made surface layer more than 50 cm thick, produced by mixing, filling, or the contamination of land surface in urban and suburban areas [10]. Several other pedologists [11,12] embraced this concept. At the end of the last century, Effland and Pouyat [13] proposed a new definition, widely accepted by both their contemporaries and current researchers [7,14]; namely, USs are “relatively unaltered soils, but subjected to urban environmental factors like atmospheric depositions”. However, this definition of USs is rather vague, and it is preferred, at present, to use the term ‘anthropogenic soil’, which broadens the concept of human-influenced soils over the previous one, which is based only on the densely human-inhabited areas [15,16]. The International Committee on Anthropogenic Soils (ICOMANTH), in different periods, introduced and modified terminologies related to anthropogenic soils, excluding any eroded (physical or chemical) soil from the definition [17]. However, since anthropogenic soils carry forward all historical information regarding the cultural practices, artifacts, and properties, anthropogenic soils can be designated as ‘golden spikes’ of the Anthropocene [18]. It is important to delve into the contemporary applications and significance of these soils in the modern environmental sciences. The study of USs has practical implications in urban planning, environmental management, and sustainable development. For instance, understanding the composition and properties of these soils is crucial for effective land use planning, pollution control, and the management of urban green spaces [19,20,21]. USs often contain a unique mix of organic and inorganic components, influenced by human activities such as industrial processes, waste disposal, and building [22]. In recent years, the concept of ’USs memory’ has gained attention. This concept refers to the idea that USs retain traces of past anthropogenic activities, which can be studied to understand the historical development of urban environments [23,24]. For instance, the characterization of deep soils may shed light on the historical progression from rural to urban activities [25]. The challenges in managing and conserving these soils are manifold. Urbanization often leads to the sealing of soil surfaces, which reduces soil biodiversity and disrupts natural soil functions. Strategies to mitigate these effects include the promotion of green areas, urban gardening, and innovative urban design, all of which provide soil conservation. These efforts not only contribute to ecosystem health, but also enhance life quality in the urban areas [26,27,28]. The study of anthropogenic/USs is a dynamic and evolving field, reflecting the changing nature of human–environment interactions. In this context, urban garden soils are important for microclimate regulation by providing shade, mitigating the maximum surface temperature, increasing the relative humidity, and allowing the infiltration of rainwater [29]. In addition, they improve air quality [30], prevent flooding by reducing surface water runoff, storing a significant amount of soil organic C (SOC), and promoting pollination by harboring several insect species [31]. Understanding, preserving, and managing sustainably USs has become vital for our planet and humanity due to the rapid increase in urbanization [10].

### 2.2. Role of Urban Soils in C Cycle and Storage

In recent decades, urbanization has intensified, resulting in a significant loss of natural soil, ultimately threatening biological diversity [32] and decreasing ecosystem services (ESs). Anthropogenic impacts in urban contexts, derived from a variety of factors, including urban construction, excavation, topsoil removal, grading, soil compaction, installation of impervious surfaces, land use typology, and environmental disturbances, severely disrupt the structure and ecological functions of soils (e.g., soil structure and stability, fertility, bioavailability of potentially toxic elements, etc.) as well as biological diversity [33,34,35]. When topsoil is removed or disturbed, a significant portion of surface organic C can be transported to aquatic systems. Microbial activity contributes to the decomposition of previously protected organic material, leading to the release of CO_2_ into the atmosphere and reducing soil C content. Then, during the urban construction phases, the natural cycle of C can be impaired, further diminishing the potential for long-term storage.

US properties and functions can differ significantly from natural soils due to the influence exerted by anthropogenic activities on short-term pedogenetic processes. For example, USs may either contain significant amounts of artifacts and human-made materials, be sealed by technic hard rock, include low-quality soil, be paved with its underlying unconsolidated materials, or be proofed with geomembranes [36]. Such disturbances can directly affect the amount of C that can be stored in the soil.

**Figure 1:**
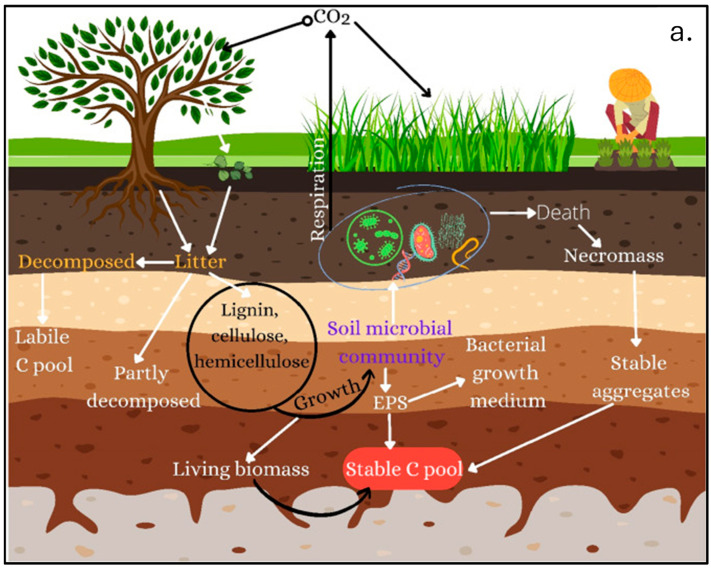

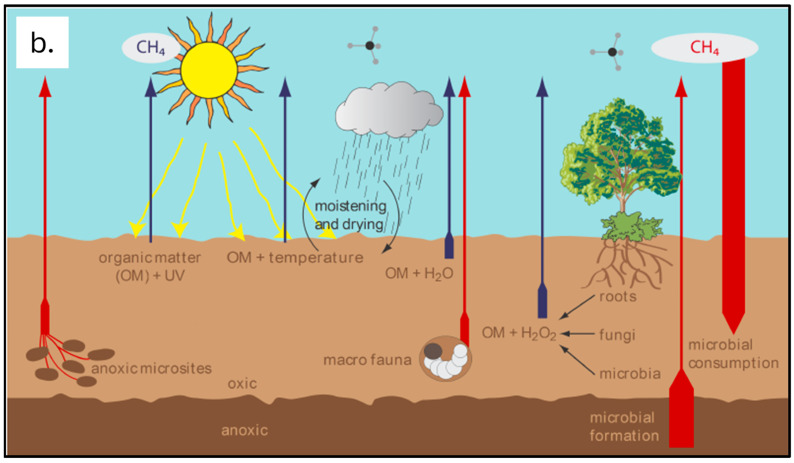
Main pathways of C cycle ((**a**) reproduced from [54] with permission from the authors) and CH_4_ transformations ((**b**) reprinted from [55] with permission from the authors) in soils.

Indeed, soil compaction, also resulting from both management practices and the recreational use of urban green spaces [37], can reduce macroporosity, which is necessary for water infiltration and drainage as well as root growth and microbial activity. This negatively affects plants, aerobic microorganisms, and overall fertility, thus causing a loss of OM content and reducing soil biodiversity [38,39]. Soil compaction also promotes waterlogging conditions, triggering the anaerobic decomposition of organic matter accompanied by the emission of CH_4_. The role of USs as a tool for C storage is often underestimated because their quality is mistakenly considered too compromised. Moreover, several studies on C sequestration in urban areas have based their conclusions exclusively on the net primary production of urban vegetation by ignoring all the contributions of the soil compartment. This notwithstanding, it has been proven that soils may provide a paramount contribution to the global C cycle and storage [40]. To this regard, in Figure 1, the main pathways of the C cycle in the USs are shown, accounting for over 70% of CO_2_ emissions from fossil fuel combustion, thus significantly implying a C sequestration, which, together with urban dwellers, manage approximately 22% of global land C intake and 24% of terrestrial C emissions. Urbanization has led to the creation of buildings and landfills, which store about 1.6% of the total global plant and soil C pools [40]. USs are thus essential to produce important ESs such as C sequestration, storage, and cycle [41]. Urban topsoils, especially those covered by vigorous vegetation, can act as C sinks [42] by capturing significant quantities of OC at contents even more abundant than those found in regional agricultural soils at equivalent depths [43]. The soil microbiota, essential in the C cycle, are notably impacted by urbanization. In densely populated areas, significant changes in microbial richness have been observed, including a decline in soil fungal communities, critical for OM decomposition processes [44].

In recent decades, particular attention has also been given to urban heat islands (UHIs), a phenomenon characterized by an increase in the temperatures of the city land surface with respect to the surrounding rural areas and prevalently caused by the presence of extended interfaces of impervious surfaces [45]. Therefore, along urbanization gradients, differences in C balance, related to CO_2_ uptake (production) and emission (soil respiration/decomposition), have often been reported [46]. The UHI effect further influences microbial respiration, posing a risk to the C stored in USs [47]. High concentrations of both inorganic (e.g., heavy metals) and organic (e.g., polycyclic aromatic hydrocarbons) pollutants in USs can also hinder plant growth [48], thereby affecting plant-driven C sequestration. Interestingly, a significant portion of urban C soil is constituted by black soil, namely derived from fossil fuels, with a longer cycle than the one present in plant litter [49,50]. The expansion of urban areas, at the expense of rural land, often leads to an initial decrease in soil C, primarily due to land use changes [51]. For instance, the urbanization of agricultural areas in China’s northeastern coastal region and the Chengdu megacity resulted in a decrease in SOC stocks by up to 13.5% [52,53], attributable to the development of infrastructures such as buildings, road-altering soil characteristics and, consequently, their properties [42]. These observations underscore the importance of sustainable urban planning and policy in climate change mitigation, focusing on the management of USs and vegetation cover to enhance C storage.

### 2.3. C Emissions and Sequestration by USs

Urban parks and green areas store as much C in their soils as either natural or rural areas near cities [56]. The C composing the SOM will remain sequestered in the soil until its mineralization is mediated by microorganisms. In particular, soil bacteria have the greatest influence on the amount of C stored in urban green areas [57]. However, the removal of OM such as grass clippings, deadwood, and leaf litter for urban green space management can have an impact on microbial activity and soil C, because this can determine the deprivation of the most important source of labile and recalcitrant OM [34]. Therefore, proper urban green space management becomes critical for conserving and increasing soil C [37,58] while also expanding urban vegetation. Moreover, anthropogenic inputs contribute to a legacy C store in USs in areas with an industrial history, significantly increasing the US C budgets. The careful management of USs can result in greater C stocks than identical soils in rural settings [42,59,60,61]. OM supplements, commonly employed in urban garden cultivation, not only increase soil C but also help to reduce greenhouse gas emissions [62]. However, economic growth, changes in land use patterns, disparities in urban architecture, and international trade, as a whole, have an impact on C emissions. These factors, in cities, are generally driven by the use of energy fossil sources, industrial production processes, and waste disposal [63].

It is very important to promote biological fertility in USs, making them more effective at sequestering C and proving stabilized OM to be exogenous. In fact, this approach may lead to various advantages with benefits for environmental and human health, such as the following:

- Climate change mitigation: USs store C in the ground, preventing it from escaping into the atmosphere [59], thus reducing the input of climate-altering gases [58];
- The mitigation of the UHI effect: the regulation and mitigation of urban area temperatures have an impact of UHI and also on CO_2_ emissions, which in turn limits energy consumption [64];
- Soil health improvement: the addition of organic amendments (e.g., compost, biochar, etc.) to USs can trigger a decades-long chain reaction of C sequestration, enhancing soil fertility and its productivity [65];
- Enhanced urban life quality: beyond being potential C sinks, both plant cover and USs provide several other ES such as enhanced stormwater management or recreational spaces for residents, consequently improving the quality of life in cities [66].

The key mechanisms that contribute to C storage in USs include a wide range of processes which are primarily driven by the dynamic interplay among plants, microorganisms, and soil characteristics. Photosynthesis and chemosynthesis are essential in this context, with autotrophic organisms serving as the primary source of SOC [67]. Producer-stored C is integrated into the soil matrix in the form of plant roots, decaying plant debris, and other organic inputs. This OM influx stands out as a critical factor underlying soil C storage [67,68]. Soil saprotrophs, including fungi and bacteria, play a pivotal role in the biological complex processes of the decomposition and transformation of OM. Their activities significantly influence the storage and stabilization of C within the soil matrix [67,69]. Furthermore, the intrinsic features of the soil, such as texture, structure, and mineral content, have a significant impact on C dynamics. Soils with higher clay content, for instance, show higher C sequestration capabilities than sandy soils due to (1) the larger surface area of clay small particles which determines stronger binding affinity to OM and protects it from its quick breakdown [70,71], and (2) the typically lower aeration of these soils which slows down aerobic OM decomposition. A well-planned urban development, when accompanied by an increase in vegetation cover, emerges as a facilitator for SOC storage. Urban vegetation cover not only contributes to C sequestration but also adds OM (such as plant litter and root exudates) to the soil, further enhancing C storage capacities [42]. The data regarding C sequestration by USs varies depending on the specific area, soil properties, vegetation, and green management practices. Although several studies examine C sequestration by USs, only a few of them have quantified it, revealing intriguing findings. For example, in Guangzhou City, China, looking at SOC storage in urban green spaces at depths ranging from 0 to 20 cm, its storage showed an average value of 2.59 ± 1.31 kg m^−2^ [68]. In another study, on a worldwide basis, urban parks appeared to contain a comparable amount of C [58]. It has been reported that the topsoil acts as a C sink due to increased vegetation, with an increment, over 30 years, of about 100% for SOC in highly urbanized cities as compared to just about 17% in the suburban areas [42]. Conversely, according to a study conducted in the United Kingdom, SOC storage, in arable soils up to a depth of 1.0 m was 14.3 kg m^−2^, was much lower than the 20.2 kg m^−2^ measured in USs [43]. Finally, a study conducted in Tacoma (USA) suggested that the application of urban organic residuals (including municipal biosolids and woody debris) to pervious surfaces could result in a C sequestration rate of 0.22 Mg C ha^−1^ year^−1^, comparable to rates reported in no-till agriculture [59]. Table 1 provides an overview of the various ways urban soils can store C, emphasizing the importance of careful management and the integration of organic materials to enhance soil health and C sequestration capacity.

### 2.4. C Emission and Sequestration by USs of Mediterranean Area

**Figure 2:**
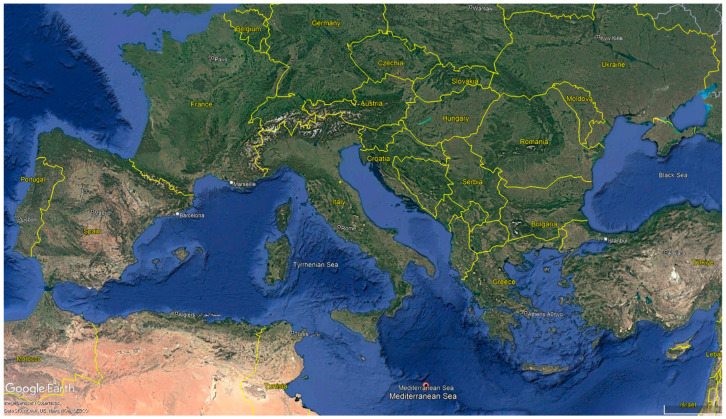
Map highlighting the Mediterranean region.

Due to its extensive area, peculiar physical and biological characteristics, and intricate geological and anthropological history, the Mediterranean area (Figure 2) poses considerable challenges for scientists and land managers.

Problems affecting soil fertility are particularly relevant in this area, characterized by high temperatures and relative moisture, which increase the metabolic activity of edaphic microorganisms (e.g., bacteria, fungi, etc.), hastening matter cycling and consuming OM faster than its accumulation [72]. However, the issue is more imprinted from a global perspective rather than of a specific geographical area [73], even though the peculiar climate characteristics of the Mediterranean basin would require a tailored approach. Overall, in relation to anthropogenic pressure, USs of the Mediterranean area [74] have low stability (in terms of both tolerance and resilience) which make them particularly vulnerable to several stresses related to pollution, climate change, drought, and desertification phenomena [75]. A recent study, presenting an outstanding approach for the optimization of urban green infrastructures (UGIs) in Granada (a medium-sized city in Spain), illustrates how cities in the Mediterranean region have great potential for green infrastructure development [76]. By considering both the availability and accessibility of green spaces, the proposed framework aims to increase urban resilience to climate change-related extreme events through strategic distribution, emphasizing the ES and prioritizing the well-being of the city inhabitants. The data demonstrate a significant increase in green spaces, offering valuable opportunities for decision-making that prioritizes equitable distribution of these areas. Surprisingly, even smaller, less active areas contribute to the reduction in UGI fragmentation, ultimately improving overall their usability, availability, and accessibility. This transformation would lead to a more sustainable and resilient urban landscape. Apart from several global-scale studies, there is a lack of research and statistically relevant data focusing on the capacity of Mediterranean USs to sequester and store C. Hereinafter, we have summarized and discussed the most relevant findings dealing with this topic. A study in Andalucia (Spain) evaluated the impact of different land uses considering their effective capability to enhance soil C storage, also in relation to sustainable management practices such as rotational grazing and conservation agriculture [77]. The authors proved that the amount of SOC stored in the grasslands varied depending on several factors (e.g., soil type, climate, and management techniques). A recent investigation examining the impact of soil sealing on potential C sequestration in functional urban areas across Europe also involved the city of Padua (Italy). This study investigated the potential of urban riparian ecosystems and afforestation as effective approaches for counteracting climate change and achieving the set mitigation goal [78]. The researchers adopted high-resolution aerial images and InVEST models to evaluate the land use/cover changes (considering the years 1955, 1981, and 2018) in the riparian ecosystems of Padua, mapping and estimating the effectiveness of C sequestration along time. Additionally, they modeled afforestation and C sequestration future scenarios from 2022 to 2050 to evaluate the potential contribution to reduce air CO_2_. Their study revealed a negative C sequestration in the urban riparian ecosystems (−928 Mg C) in the period from 1955 to 2018. However, they estimated that C stocks might increase by about 4300 Mg C in a future scenario (2022–2050), contributing towards city C neutrality. Canedoli et al. (2020) showed that USs in Milano (Italy) have a great potential to stock C, with a mean value of SOC of about 20 g kg^−1^. Gratani and Varone (2005) showed that in correspondence with urban parks in the city of Rome (Italy), CO_2_ concentration decreased by about 3.5% from the first hours of daytime to 11.00 a.m. due to the higher photosynthetic activity during those hours, underlying the importance of planning and developing green urban areas. Despite several studies qualitatively discussing the benefits of greenery and USs in areas, particularly in the Mediterranean, the scientific literature is lacking in publications that highlight the quantification of these benefits for the urban population. Therefore, scientific papers demonstrating and quantifying the benefits resulting from greenery [37] and the proper management of USs could encourage and stimulate the establishment of greener cities.

## 3. Plant Communities as Modulators of C in Urban Areas

Gaining insight into the potential advantages of plant communities when designing cities and planning sustainable urban development becomes relevant for enhancing both environmental and human well-being. In anthropogenic/urban soils, all urbanization-related aspects affect both epigeal and underground structures and the functions of plant communities. Anthropogenic activities heavily influence latent heat flux and relative vegetation, causing considerable variations both in plant phenology and functional traits associated with community structure [79]. Soil compaction, enhanced by the presence of impervious surfaces, limits plant root systems, fostering the selection of a particular and unique plant community within the urban contexts [21,80]. Furthermore, either the alkalization or acidification of urban soils, due to meteorological agents, as well as high temperatures, evaporation rates, and low soil moisture, foster tolerant plant species to those environmental factors at the expense of native vegetation [42,81,82]. It has been shown that a substantial role in plant community composition, richness, and evenness in urban areas is exerted by land use, which determines ecological niche partitioning. For instance, in urban microhabitats, a significant shift in the diversity of plant species has been reported for Asteraceae and Poaceae families, associated with changes in the allocation of functional traits, particularly in terms of biological growth forms [83]. Notably, inside urban settings, therophytes tend to dominate over geophytes or phanerophytes [84]. In fact, invasive alien and cosmopolite plants, as well as ruderal species (which have both great efficient reproductive fitness and good capacity to exploit environmental resources) can better colonize degraded and marginal soils in urban areas than native vegetation [20,84,85]. The environmental conditions of abandoned areas, roads, and ditch margins (e.g., higher temperatures, acidity, and less fertile soils with low available nutrients) determine the establishment of a hostile environment where invasive alien plants are more favored than the autochthonous ones [86]. The heterogeneous spatial configuration of urban ecosystems, composed of a hostile matrix of impervious surfaces in which few green spaces with altered soils are present, determines a reduction in plant biomass production caused by water stress and differences in C uptake and storage [87,88]. Altered environmental conditions, because of the multiple anthropogenic activities reflecting in either higher CO_2_ concentrations or nitrogen availability, might improve vegetation productivity at the expense of an increase in plant mortality rates [89]. Management practices, such as mowing and the recreational use of green areas, are among the major drivers in determining plant community structure [90]. However, repetitive mowing reduces plant above-ground biomass without influencing species richness, resulting in a positive effect in increasing plant biodiversity by preventing rapid successions which would cause the loss of less abundant species as well as the dominance of perennial grasses [91]. In this context, mowing would increase light availability for low-statured and subdominant species with the result of fostering their seeds’ germination to the disadvantage of plants with wide, lignified root systems [68], causing a more rapid turnover of opportunistic plant species. As an effect of habitat heterogeneity, which hosts a variety of microhabitats (such as walls, ruins, stones, sidewalks, railways, etc.), urban ecosystems usually show high vascular plant richness, which can foster the biodiversity of other edaphic taxonomic groups. Based on these observations, vegetation may increase urban ecosystems’ resistance and resilience, even by mitigating the impacts of the disadvantages related to these environments [92]. When urban planning is addressed in terms of more sustainable development toward greener cities, vegetation and related ecosystems become a source of a wide range of important services [93] such as CO_2_ sequestration, cooling UHI temperatures, improving air quality, and environmental healthiness [94]. Plants play a vital role in maintaining ecosystem stability and functioning since they regulate several important processes both in the C cycle and in modulating climate and microclimate conditions and much more [90,95]. This applies not only to herbaceous plants but also to shrubs and trees [96]. In fact, attention is paid to urban parks (and even more to urban forests) whose ecosystem services can be estimated by land cover analyses through satellite remote sensing [97,98,99]. One of the possible applications of these methods is the evaluation of C sequestration. Even though CO_2_ fluxes in urban areas are mainly determined by anthropogenic emissions (e.g., traffic, domestic heating, industrial processes, etc.), large green areas, such as urban parks and boulevards, can greatly contribute to C sequestration [100,101]. This process depends on vegetation characteristics and, in particular, if the planted species are herbaceous, shrubs or trees [96], or if they are deciduous or evergreen, acting in the last case as a C sink throughout the seasons [102]. In fact, for plant communities, C storage is clearly associated with plant growth rate and dynamics [103]. Chan et al. (2018) went deeper into these aspects, with the formulation of a dynamic ecophysiological model for various vegetation mixes in urban ecosystems, which showed that gardens with fast growing plants rich in lignin (high C:N ratio) can accumulate carbon at a higher rate than slow growing ones: quantitatively, the authors report that the C contents in herbs, shrubs, and trees were 22.6 ± 6.9%, 31.3 ± 4.0%, and 32.4 ± 7.7%, respectively (average ± standard error; Chan et al., 2018). Similarly, Whittinghill et al. (2014) highlighted that in two years of study, woody plants can store carbon at a rate ranging from 62.91 to 78.75 kg m^−2^, which is slightly more than herbaceous perennial grasses whose carbon storage reaches up to 68.75 kg m^−2^. Also, the authors calculated that other landscape systems, such as native prairie mixes and herb/vegetable gardens, can accumulate a moderate amount of C compared to the vegetation that was formerly in their place (11.03 and 54.18 kg m^−2^, respectively), although these values appear to be very variable in above-ground and below-ground biomasses because of the effects of shallow soils in urban environments [104]. Despite the fact that the contribution of each urban lawn or garden in carbon storage is small, from a cityscape point of view, a comprehensive connected system of urban green areas can provide bottom-up scalability and contribute to ecosystem services, including C storage [105].

Urban parks can mitigate the temperatures caused by soil sealing, especially in the Mediterranean area, where both deciduous and evergreen species could provide thermal comfort; thus, specific expertise in managing these areas is strongly required [102,106,107]. In this contest, city climate/temperature regulation has become one of the major challenges for decision-makers, as confirmed by the fact that in several Mediterranean areas, the rate of human mortality due to heat waves has significantly increased during the last 10 years [93]. Several studies show that the tree cover of the green areas can significantly reduce the temperatures of the urbanized areas, with positive spill-over effects in the surroundings [108]. Mediterranean areas are typical environments hosting unique vegetation, with its history reflected in soil characteristics, in terms of functioning and organic C stock [109]. Gratani et al. (2016) [102] highlighted that the amount of carbon stored in four urban parks in Rome is equal to 319.7 kg CO_2_ m^−1^ year^−1^, while individually, each park contributed to 79.9 ± 12.3 kg CO_2_ m^−1^ year^−1^. Overall, to enhance the capability of urban green areas to provide ecosystem services, the main strategies proposed by different authors [110,111,112,113] are the reconversion of anthropized areas to natural environments and the implantation of vertical greenery, together with the increase in the connectivity of the urban tissue and the quality of the green patches. A high density and diversity of green infrastructures in the mosaic of urban matrix was proved the best scenario for the development of urban settlements. A case study was proposed by Fasolino et al. (2023) [37], in which two scenarios composed by different vegetation communities (different mix of trees and bushes) were compared: the first case with more trees than bushes and the second one the other way around. The study highlighted that in the former some features such as wind attenuation and connectivity indexes were significantly higher than the latter, thus implying that plant community composition and structure can improve the overall health of urban environments through sustainable urban planning [37]. A more comprehensive work was recently given by [114]. The results obtained by these two authors can be summarized as follows: (i) urban forests are usually studied more than urban parks (there are more than double the amount of papers regarding urban forests than ones regarding urban parks); (ii) interestingly, the number of papers concerning urban parks increased in the period from 2002 to 2023, and (iii) the main areas involving the study of the provision ecosystem services of green infrastructure are equally represented by natural sciences and social sciences [114]. Ultimately, understanding the potential benefits of vegetation in fostering sustainable urban development and its planning becomes of paramount importance to improve both environmental and also human health [37].

## 4. Soil Microbiomes as Modulators of C in Urban Soils

Most studies concerning soil microbial ecology primarily focus on rural regions [115]. Soil microorganisms play a crucial role in both the input of C into the soil and the output of CO_2_ into the atmosphere. In fact, on the one hand, they can contribute to C sequestration in soil via photosynthetic microorganisms and C immobilization in microbial biomass [116]. On the other hand, the heterotrophic respiration of soil microorganisms leads to a significant soil-derived exhalation of CO_2_ into the atmosphere, which exceeds the amount of CO_2_ emitted by fossil fuel combustion by a factor of ten [117]. Pappalardo et al. [118] underscored the pivotal functions of soil microorganisms, not only in the decomposition process, which results in organic C loss, but also in the formation and long-term retention of SOC. This is evident through the interrelation among microbial biomass, necromass, and SOC content [119,120,121,122].

**Figure 3:**
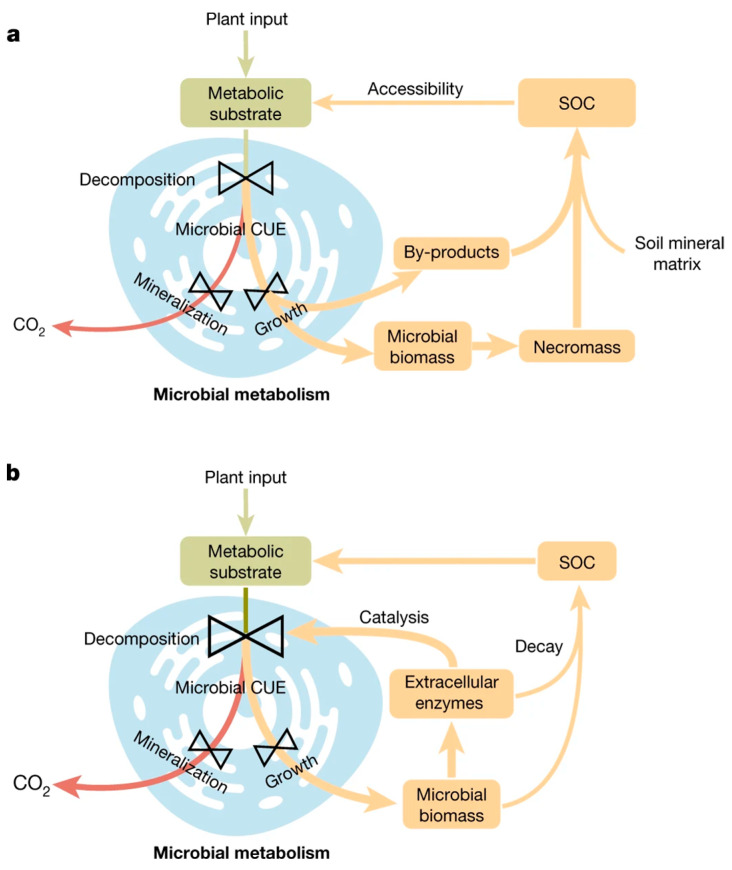
Two divergent routes (**a**,**b**) influencing the connection with microbial C use efficiency (CUE), promoting global soil C storage and soil organic C (SOC) storage. Microbial CUE and microbial C uptake (ng C g^−1^ h^−1^) can be calculated by the following equation [126]: (CUE = C growth/C growth + C respiration = C growth/C uptake). Reproduced from [127] with permission from the authors.

Two divergent pathways influence microbial C use efficiency (CUE) (Figure 3). The first pathway suggests that a high microbial CUE promotes the buildup of SOC storage by fostering a greater amount of microbial biomass and the production of associated by-products. The second pathway underscores that a heightened microbial CUE triggers soil SOC losses through the augmentation of microbial biomass and the subsequent production of extracellular enzymes, thereby enhancing SOC decomposition [80,123]. Consequently, even minor alterations in the soil C cycle could exert a significant effect on atmospheric CO_2_ concentrations [118]. The diversified microbial communities in urban soils offer numerous valuable services in the cities. Nevertheless, there is a lack of exhaustive information regarding the ecology of microbial communities in urban soils, necessitating further studies to enhance our ability to predict the impact of urban land use [124]. The study by Tian and Jing (2024) [125] delves into the pivotal function of soil microbes in the global C cycle and critically evaluates various methods to pinpoint the microorganisms responsible for C inputs into the soil. The conclusion drawn is that, while these technologies are essential in identifying C-consuming microbes, they fail to deliver a high-capacity quantitative analysis of C usage based on phylogenetic groups. Moreover, the considered methodologies cannot exhaustively capture CUE and its ultimate destination within the microbial metabolome.

### 4.1. The Role and Abundance of Microorganisms in Urban Soil and Atmospheric C Sequestration

**Figure 4:**
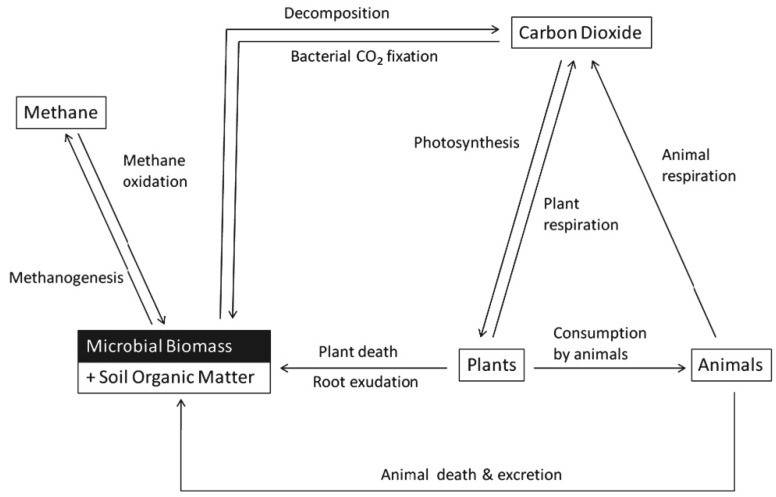
The terrestrial C cycle with the major processes mediated by soil microorganisms. Reproduced from [133] with permission from the authors.

Microbial metabolites and enzymes are important precursors of the humification process, thus contributing to the naturally occurring development of a biochemically stable SOM. Comprising several thousand species per gram of soil, microorganisms represent a substantial portion of soil biodiversity. At the same time, they play a crucial role in regulating essential processes, such as the decomposition of OM and nutrient cycling [128,129]. Extracellular microbial C also influences the stability of SOC. Several soil microbes are known for their C fixation and storage ability (e.g., Rhizobia, Actinobacteria, Cyanobacteria, etc.) [118]. Soil microbial C biomass, hereafter referred to as “microbial C”, defines the C content within bacterial and fungal cells per unit of dry soil. It serves as a valuable metric for assessing the size/dimension of the microbial community [130], estimated through a set of techniques developed since the 1970s, including fumigation, substrate-induced respiration, and the analysis of phospholipid-derived fatty acids [131]. Specifically, microorganisms play a crucial role in SOM decomposition, regulating soil C reservoirs (Figure 4). Throughout this process, they can either immobilize the absorbed C for the production of new biomass, or release it through respiration, in the form of atmospheric CO_2_. The ratio of growth to overall assimilation establishes the microbial C use efficiency [132]. Consequently, CUE holds significant implications for the impact of microbial decomposers on the equilibrium of C distribution between soil and atmosphere. CUE stands out as a factor of at least fourfold significance compared to other assessed variables, including C input, decomposition rate, or vertical transport, when it comes to influencing the storage of SOC and its distribution worldwide, and there is a positive relation between CUE and SOC content [123]. Several studies have revealed that fungi can exhibit significant activity in soils rich in high-quality C, even when it is present in low quantities [132]. Recent investigations employing ^13^C-labeled substrates have shown that soil fungi play an important role not only in breaking down complex compounds but also in the decomposition of relatively easily degradable plant material. In fact, fungi utilize C fractions, easily degradable, present both in the rhizosphere and during the initial phases of plant litter decomposition [133].

Overall, soil microbial communities are considered the most important quality indicators, often surpassing physical and chemical properties [134]. They are influenced by the interplay of both geochemical and biological processes by plants, specific environmental conditions, as well as urbanization. Among these factors, the plant rhizosphere provides a specific microhabitat where complex interactions occur, selecting microbial populations well adapted to this unique niche. Accordingly, the urbanization factors influencing rhizosphere properties may also induce compositional shifts in microbial communities [135]. In this context, recent studies have indicated that urban ecosystems and high soil heterogeneity contribute to a significant variability in the microbial metabolism associated with respiration and enzymatic activities [136], as well as to their abundance when compared to natural or agricultural soils. This complexity makes it challenging to generalize observed patterns and develop a predictive understanding of their dynamics [137]. Consistent with previous studies, conducted across various locations and soil types, Proteobacteria, Acidobacteria, Actinobacteria, and Verrucomicrobia have been identified as predominant phyla of the urban soil microbiome [138]. Soil surface excavation, compression, and several other kinds of human alterations exerted on urban environments lead to the soil’s deterioration in quality even from the microbiological point of view and, consequently, of multiple services provided by soil ecosystems. Acknowledging these threats, it is imperative to transition towards novel models of urban development and management based on the concept of “reintroducing nature to cities” [115]. In this context, the supply of exogenous organic products, hopefully deriving from the valorization of waste materials, represents a virtuous and sustainable strategy to restore urban areas and enhance the quality of these compromised soils [125]. Several studies have shown that organic amendments are able to restore degraded soils, but their influence on the structure of the microbial community in urban soils is still poorly known [34]. In fact, there is still a knowledge gap about the representative bacterial taxa useful for the ecological restoration of degraded soils to bring them back to their natural state [34]. While urban soils are a relevant part of green spaces in the cities, they are not adequately studied, especially in the Mediterranean area, where unique climate conditions and factors like land use, pollution, and vegetation cover, significantly influence microbial activity, carbon storage, and carbon fluxes. Nevertheless, some key studies on urban soils of Mediterranean area shed light on how the soil microbiome influences C dynamics in these soils. In urban soils in southern Italy, microbial biomass C ranged from 0.6 a 3.6 mg C g^−1^ soil, with higher values under holm oak vegetation cover. The higher values correlated with greater organic matter content and more active C cycling [35]. Microbial community diversity and the composition of urban soils can affect the rate of C sequestration and the turnover efficiency. C use efficiency and SOC stocks reached the highest values in urban parks and gardens compared to more disturbed soils (e.g., industrial and commercial zones). The average SOC of urban parks in Milan was higher (7.9 ± 2.4 kg m^−2^) than urban non-parks (5.3 ± 2.5 kg m^−2^), while SOC stock did not significantly differ for urban land cover types [139]. These differences can be attributed to the variation in microbial activity and to more efficient carbon conversion processes and organic matter input across different land uses.

It is noteworthy that the application of organic amendments improves soil quality in both agricultural [140,141,142] and urban settings. The application of organic amendments may change the activity and diversity of soil microbial communities. In fact, a long-term study (25 years of data collection) reported that the application of soil amendments, derived from organic waste, strongly influenced the structure and functionality of microbial communities [143]. Compost, derived from domestic solid waste and applied to Mediterranean degraded soils, favored, in the long-term period, greater microbial diversity and the proliferation of several bacterial populations abundant in natural soils (e.g., *Craurococcus*, *Phaselicystis*, *Crossiella*, etc.), contributing to the formation of a bacterial co-occurrence pattern [134]. Other studies reported a significant increase in fungal biomass and a decrease in microbial community stress after biosolid and plant yard waste compost treatments on degraded soils [125]. Similarly, the addition of other organic fertilizers (pig manure and slurry) caused changes in the microbial community structure [144]. The use of biochar has been shown to increase the number of fungi and Gram-negative bacteria, as well as the proportion of Actinobacteria [145] in several different soils. The main driving force of microbiota is the high heterogeneity of urban ecosystem soils, where microbial activity is largely influenced by the presence of SOM [138]. Moreover, it becomes possible to achieve highly efficient C fixation by applying organic amendments selected based on specific properties and characteristics, and/or potentiated with functional materials [134].

### 4.2. Identification and Characterization of Urban Soil Microorganisms

To understand how microbial communities contribute to the overall functioning of the soil ecosystem [137], it is possible to describe their structural or functional biodiversity and apply new synthetic indices [146]. Earlier indications of transient shifts in microbial community composition, following organic amendments, primarily relied on denaturing gradient gel electrophoresis [147]. Likewise, only studies employing molecular methodologies to analyze nucleic acids (DNA or RNA) in soil extracts, for investigating the diversity of microbial communities, are now taken into consideration. These approaches are deemed the most decisive and promising for characterizing and monitoring the diversity of these communities in various urban systems [148]. Nevertheless, a comprehensive genomic representation of microbial variation in soils subjected to prolonged remediation remains elusive, and several researchers (see, e.g., [149]) have asserted the necessity for extended analytic studies. Such studies are crucial to grasping the abiotic and biotic factors influenced by SOM, ultimately enhancing our ability to predict how ecosystem functionality responds to global change. However, the intricacy of microbial diversity is not trivial, with one gram of soil potentially harboring billions of microorganisms from thousands of distinct species. Currently, next-generation sequencing (NGS) is offering a deep insight into the composition of soil microorganism communities. The advent and continuous enhancement of high-throughput sequencing platforms have significantly advanced the insight into complex microbiomes and microbiota in both bulk soil and the rhizosphere. Over the past decade, DNA metabarcoding has swiftly emerged as a powerful and cost-effective technique for describing microbiomes in environmental samples. However, this approach entails several processing steps, each of which may introduce notable biases that could significantly compromise the reliability of the metabarcoding results, but currently DNA metabarcoding stands as the most widely employed molecular approach for characterizing microbiomes in environmental matrices. QIIME and MOTHUR are the predominant platforms employed for bioinformatic analyses of the metabarcoding data [148]. Despite its usefulness, there is still a restricted understanding of how shifts in microbial community compositions correlate at the genomic level with metabolic functionality and soil quality [143]. In a study on phylogenetic and functional modifications in the microbial community of soils subjected to long-term restoration, the pyrosequencing of 16S- and 18S-rRNA genes did not reveal significant differences in phylogenetic diversity among restored and control soils [150]. In the long-term, soil restoration under semiarid conditions did not increase microbial diversity but influenced microbial community structure and functionality [143].

## 5. Impacts of Strategies and Management Practices on C Stock in Urban Soils

In urban areas, soil amendments are used to support local food production policies, maintain vegetation for landscape and recreational use, and rehabilitate degraded soils [151]. However, studies investigating the application of soil amendments in urban settings remain limited as compared to those describing the same applications in agricultural soils. While in the past the use of soil amendments aimed at improving soil fertility and plant production, only recently has it has been recognized that these amendments can also play a relevant role in mitigating the effects of climate change [152]. Indeed, they have a substantial role in effectively sequestering C by retaining a greater amount of it, compared to the roles present either in the atmosphere or terrestrial biomass [153]. Conventional soil management practices, also in urban contexts, involve the addition of bioavailable nutrients in the form of mineral fertilizers. The amendment through organic fertilizers, used as a common agricultural practice for thousands of years in the past, is a well-established alternative to mineral fertilization. Organic amendments, when compared with mineral fertilizers, have a long-term beneficial effect, due to both the slow release of nutrients and the presence of more stable C. According to the International Biochar Initiative, biochar is charcoal largely used as a soil amendment to increase fertility and ensure a long-term C storage. Specifically, biochar is a C-rich material organized in aromatic structures and occasional heaps of graphite-like layers. Due to these properties, when applied to the soil, it is expected to remain stable for many years (100 to 4000 years), as approximately 80–95% of it is highly resistant to microbial decay and improves C storage in the soil (Nogués et al., 2023) [154]. In fact, Moreno et al. (2022) reported an increase in TOC of 10–30 g kg^−1^ after an application of 100 t ha^−1^ (20 t ha^−1^ year^−1^) [155] and Sánchez-Monedero et al. (2019) reported an increase of about 80% in TOC after two and three years, respectively, applying a total of 40 t ha^−1^ of biochar [155,156]. A meta-analysis of several measurements, coupled with peer-reviewed articles, showed that biochar application was the most effective organic amendment to increase SOC content (by an average of 39%) compared to plant cover (6%), or conservation tillage (5%) [157]. However, the biochar C soil addition is not completely inert and can be slowly mineralized through biotic and abiotic processes. In fact, several studies report that only the labile C of the biochar is employed by microbiota [156]. While the effects of biochar application on soil chemical properties are widely known [158], research on how its physical and biological properties affect soil is still limited. In this context, the pyrolysis temperature of the starting material for biochar production is very important. Benavente et al. (2018) [159] studied the effect of three different biochars obtained from the pyrolysis of the organic fraction of municipal solid waste at different temperatures (300 °C and 500 °C) and residence times (1 h and 5 h) on the biochemical properties of agricultural soil, by monitoring CO_2_ emissions, microbial biomass, and the enzymes dehydrogenase, phosphomonoesterase, and β-glucosidase. Specifically, two types of biochar produced at 300 °C but with two different residence times (i.e., one hour and five hours), and one biochar obtained at 500 °C with a residence time of one hour, were tested as soil improvers. A phytotoxicity test was performed and the two biochars, produced at 300 °C, determined lower germination values (attributable to the higher bioavailability of heavy metals and solubility of OM), while the biochar, prepared at 500 °C, caused a relevant phytostimulatory effect. Moreover, the two types of biochar produced at 300 °C increased CO_2_ emissions from the soil. Microbial biomass, phosphomonoesterase, and dehydrogenase activities were double in the case of soils amended with biochar prepared at 300 °C (regardless of residence time), while β-glucosidase decreased in all treatments compared with the control. Zabaniotou et al. (2020) [160], from a literature screening, found that the application of biochar, with standard characteristics and produced from organic waste suitable for Mediterranean conditions, enhanced soil fertility, improved urban air quality, and positively contributed to climate change mitigation. Biochar is typically characterized by a relatively large number of functional groups developing pH-dependent charges that are mostly negative for soils with neutral and alkaline pH (high cation exchangeable capacity, CEC). On this basis, its application favored the retention of several cationic nutrients through ionic adsorption, making them bioavailable for urban plants and soil microorganisms, and, partially, satisfying the demand for mineral fertilizers. Importantly, the conversion of a waste feedstock into a biochar occurs through pyrolysis which leads to condensed forms of C rather than decarboxylation processes (resulting in the release of CO_2_). Therefore, biochar soil application considerably reduces the direct and indirect release of CO_2_ into the atmosphere, contributing positively, together with the oxygenic photosynthesis (deriving from plant growth promotion), to balancing its emissions. As a widely used organic soil amendment, compost is able to improve soil quality in terms of chemical, physical, and biological properties [161,162,163]. It can be effectively applied to restore degraded urban soils by alleviating erosion pressure and improving the stability of soil aggregates, especially in clay and silty soils, and thus promotes micro- and macro-agglomeration through the flocculation of the soil particles [164]. It also reduces bulk soil density at soil depths of 0–15 and 15–30 cm compared with mineral fertilizers by improving soil structure [165]. Although it highly depends on both the composting method and initial biomass composition, compost is generally characterized by a neutral or slightly alkaline pH and, to a certain extent, could be used as a soil pH conditioner [166]. In addition, compost greatly contributes to the growth and functioning of beneficial soil microorganisms because it not only introduces thousands of favorable microbial species but also stimulates their activity, with particular concern to nutrient cycling [167]. In this respect, compost contributes to increasing the content of SOM, providing, through a slow-release, essential nutrients such as N, P, and S, and enhancing the soil CEC. Erhart and coworkers [168] also pointed out that compost has the potential to sequester C and reduce greenhouse gas emissions, although this potential decreases when soil is saturated by C. In fact, the authors state that the annual application of 8 t ha^−1^ of compost produces a positive balance of 115 kg C ha^−1^, the annual application of 14 and 20 t ha^−1^ of compost produces a positive balance of 1021 kg C ha^−1^, while the annual mineral fertilization of 29–62 kg N ha^−1^ results in a negative annual balance (−169 to −227 kg C ha^−1^). However, increased soil greenhouse gas emissions have been associated with the use of compost, which could be related to its quality and soil pedological features [169]. Moreover, differently than for biochar production, it is important to underline that the composting process involves decarboxylation and respiration steps, which release a non-negligible amount of CO_2_ during its production process. According to Lynch et al. (2006) [170], 75–95% of organic C applied with compost soil amendment increases OM recalcitrance with prolonged storage in non-mineral soil fractions even one year after its addition. However, concerns arise about the long-term use of organic fertilizers, with the possible accumulation of non-essential elements in the soil and, consequently, their transmission to the food chain and finally to humans. In this context, a long-term study published by Baldantoni and coworkers [171] looked at the total concentrations and bioavailability of potentially toxic elements in soils fertilized with the minerals or with compost obtained from organic waste, or with a mixture of the two. They demonstrated that soil compost amendment, alone or in combination with mineral fertilizers, reduced the bioavailability of Cu, whilst it improved the availability of K, Fe, Mn, and Zn, excluding their long-term accumulation in the soil. Concentrations of total and bioavailable fractions of non-essential elements (apart from available Cd) did not vary with treatments. Another study, conducted by Picariello and coworkers [172], showed that a soil amendment conducted with a compost obtained from organic waste had positive effects on the SOM and P concentration, as well as on microbial community functionality; meanwhile, soil amendment with a selected sewage sludge resulted in no benefit at all [172]. Moreover, repeated organic soil amendments not only increased SOM, but also stimulated the development of a luxuriant flora capable of maintaining a significant microbial load and providing a near-constant delivery of nutrients through its inputs to the soil [79]. Malone et al. (2023) [62] performed a literature review to monitor the C content of urban soil after the addition of different amendments (such as compost, biochar, and biosolids). The authors reported that, for the urban sites analyzed, the percentage of SOM increased regardless of the type of amendment employed. Interestingly, both compost and biochar resulted in a greater increase in SOM of 3.1 and 6.5%, respectively, while biosolids reported a small increase in the percentage of SOM but promoted an increase in nutrients compared to the other soil amendments. Moreover, organic soil amendments also improved the N content and decreased bulk soil density. Because of the synergistic effects of biochar and compost, their combined application has been recognized as a highly promising and effective method for soil improvement [173,174,175]. A lack of awareness and publicity about the effectiveness of combined biochar-compost action on various soils determines a limited application of this mixture. Kammann et al. (2017) reported that the biomass yield of poor soils was significantly increased by about 300% after the application of co-composted biochar, however biomass yield decreased after the application of the biochar alone [176]. Bolan et al. (2015) suggested that the combined use of compost and biochar was more effective in C stabilization and sequestration than biochar alone [177]; meanwhile, the interaction between biochar and compost, in combined applications, has received increasing attention. Biochar can extend the residence time of compost in the soil, and, at the same time, compost can solve the problem of nutrient deficiency provided by biochar. Thus, biochar-compost combination can reduce the mineralization rate of SOM and meet the vegetation high demand for nutrients. Therefore, biochar combined with compost is the most recommended mixture able to replace or, at least, reduce the use of mineral fertilizers in long-term sustainable management of either agricultural or urban soils. Biochar-compost application to the soils also effectively alleviates some of the side effects caused by water scarcity, and, substantially, reduces the threat due to a high salinity present in certain soils, often experienced in the Mediterranean area. The relationship between soil organic amendments and retention of C has been studied by several researchers [178], who have come to conflicting conclusions regarding the appropriate quality and quantity of different kinds of OM applied to the soil. Anyway, the effect of organic amendments on SOM should be studied through long-term rather than short-term field experiments. In general, the addition to soils of a grate amount of OM makes a larger fraction of the SOM susceptible to oxidation and microbial decomposition.

## Figures and Tables

**Table 1:** Key mechanisms for carbon sequestration in urban soils.

Mechanism	Description	Key References
Photosynthesis and chemosynthesis	Producers, primarily autotrophic organisms, integrate C into the soil matrix via plant roots, decaying plant debris, and other organic inputs	[67]
Soil saprotroph activity	Fungi and bacteria decompose and transform organic matter, influencing C storage and stabilization within the soil matrix	[69]
Soil texture and structure	Higher clay content in soils leads to higher C sequestration due to larger surface area and lower aeration, protecting OM from quick breakdown	[71]
Urban vegetation cover	Increases in vegetation cover contribute to C sequestration and introduce OM such as plant litter and root exudates into the soil	[42]
Organic amendments	Addition of compost, biochar, and other organic amendments enhances soil fertility and initiates long-term C sequestration	[65]
